# Drugs from the Sea: A Marine Sponge-Derived Compound Prevents Type 1 Diabetes

**DOI:** 10.1100/tsw.2001.357

**Published:** 2001-11-06

**Authors:** Luc Van Kaer

**Affiliations:** Howard Hughes Medical Institute, Department of Microbiology and Immunology, Vanderbilt University School of Medicine, Nashville, TN 37232, USA

**Keywords:** Type 1 diabetes, autoimmunity, natural killer T (NKT) cells, CD1d, glycolipids, α-galactosylceramide (α-GalCer), immunotherapy, non-obese diabetic (NOD) mice

## Abstract

More than one million Americans have Type 1 diabetes. This disease — also known as autoimmune or juvenile diabetes — strikes children suddenly, makes them dependent on insulin injections for life, and carries the constant threat of devastating complications. While it can and does strike adults, nearly half of all new cases are diagnosed in children. A child is diagnosed with Type 1 diabetes every hour. Type 1 diabetes is caused by the inability of a person’s pancreas to produce sufficient amounts of insulin to control their blood sugar levels and sustain life. While insulin injections allow affected individuals to control their blood sugar and stay alive, it is not a cure nor does it prevent the devastating complications of this disease, which include kidney failure, blindness, amputations, heart attack, and stroke. In Type 1 diabetes, the body’s own immune system goes awry, attacking and destroying insulin-producing cells in the pancreas.

Two recent papers in *Nature Medicine*[1,2] and a third paper in the *Journal of Experimental Medicine*[3] describe a potentially new therapy for Type 1 diabetes. These investigators found that a marine sponge-derived chemical, α-galactosylceramide (α-GalCer), prevents Type 1 diabetes in mice by a mechanism predicted to have a parallel in humans. When injected into mice, α-GalCer activates a group of immune cells — natural killer T (NKT) cells — that in turn suppress the pathogenic T cells that errantly attack insulin-producing cells in the pancreas.

T lymphocytes are critical mediators of the immune response against infectious agents. Although T cells normally effectively distinguish between friend and foe, they sometimes errantly react against the body's own cells. This autoimmune reaction results in chronic inflammation of the target tissue. Immunologists have divided T cells into two categories with specialized functions[4]: T helper type 1 (Th1) cells contribute to immune responses against intracellular pathogens, whereas Th2 cells are critically important for the generation of protective antibodies against extracellular pathogens. Most evidence indicates that Th1 cells promote autoimmunity, whereas Th2 cells play a regulatory role. One approach to the treatment of autoimmune diseases is to try to turn the pathological immune response into an innocuous one. To accomplish this, a variety of techniques have been employed, collectively called immune modulation. Studies in an animal model for Type 1 diabetes, the non-obese diabetic (NOD) mouse, have shown that modulation of immune responses from a Th1-dominant to a Th2-dominant response can effectively prevent disease[5]. For example, disease is prevented by injecting NOD mice with factors such as interleukin-4 (IL-4) that promote Th2 responses, or by administration of antibodies directed against Th1-promoting factors such as interferon-γ (IFN-γ).

α-GalCer is a glycolipid — a chemical containing part sugar and part fat — that was originally isolated from the marine sponge *Agelas mauritianus*, as a compound with profound antimetastatic activities in mice[6]. It was subsequently found that this natural product and its synthetic homologue activate NKT cells[7], a small population of T lymphocytes whose precise functions remain unknown[8]. Recognition of α-GalCer by NKT cells requires its interaction with the cell surface receptor CD1d. Injection of α-GalCer into mice has profound effects on immune responses mediated by conventional T cells, promoting Th2 cell responses[9,10]. Because Th2 immune responses can suppress autoimmunity the researchers hypothesized that activation of NKT cells with α-GalCer can be used as an alternative way for immune modulation of autoimmune disease. Thus, the ability of α-GalCer to modulate Type 1 diabetes in NOD mice was tested. NOD mice generate a spontaneous form of Type 1 diabetes that closely resembles the human disease[11]. In this strain, nearly 90% of female mice develop diabetes by 4–6 months of age. Injection of α-GalCer in these animals inhibited development of diabetes in a dose-dependent manner, particularly when treatment was initiated at an early age[1,2,3]. α-GalCer treatment also prolonged the survival of islet transplants into newly diabetic NOD mice[2]. To test whether these effects are mediated by NKT cells, the NOD mice were bred with a strain that lacks the CD1d molecule and shows a dramatically reduced number of NKT cells, creating a NOD strain that lacks NKT cells but still develops diabetes. Injecting α-GalCer into these mice did not prevent the disease[1], indicating that the preventive effect is NKT cell-dependent. The results further showed that disease prevention is associated with reduced IFN-γ and enhanced IL-4 production by pancreatic cell-specific T cells[1,2]. Thus, α-GalCer can effectively tip the balance from a pathogenic Th1 toward a protective Th2 cell response.

One interesting aspect of the CD1d antigen presentation system is its high degree of conservation among different species. Thus, the new studies should be directly applicable to treatment of the human disease. Although α-GalCer has significant liver toxicity in mice, no adverse effects have been observed in clinical trials for using α-GalCer as a treatment for human cancers. With regard to its clinical applications for Type 1 diabetes, α-GalCer holds most promise for preventing disease in individuals that are at risk for developing diabetes, e.g., those who have parents or siblings with the disease and have elevated blood antibody levels against pancreatic proteins. In patients where significant damage to the pancreas has already occurred, α-GalCer may be able to halt further destruction. However, this possibility will require more rigorous testing in NOD mice. In future studies it will be interesting to test the efficacy of analogs of α-GalCer — essentially the same chemical but with minor structural modifications — for treatment of Type 1 diabetes. Some of these analogs may have fewer side effects than α-GalCer itself. It will also be worthwhile to combine α-GalCer with other therapeutics, proven or in testing, to evaluate whether a combination of compounds is more effective. Finally, because Th1 cells have been implicated in many pathological conditions, α-GalCer treatment may be applicable to all autoimmune diseases such as Type 1 diabetes, multiple sclerosis, and rheumatoid arthritis, in which a Th2 bias is beneficial.
